# Enhanced Osteoclastic Resorption and Responsiveness to Mechanical Load in Gap Junction Deficient Bone

**DOI:** 10.1371/journal.pone.0023516

**Published:** 2011-08-29

**Authors:** Yue Zhang, Emmanuel M. Paul, Vikram Sathyendra, Andrew Davison, Neil Sharkey, Sarah Bronson, Sundar Srinivasan, Ted S. Gross, Henry J. Donahue

**Affiliations:** 1 Division of Musculoskeletal Sciences, Department of Orthopaedics and Rehabilitation, Penn State College of Medicine, Hershey, Pennsylvania, United States of America; 2 Department of Cellular and Molecular Physiology, Penn State College of Medicine, Hershey, Pennsylvania, United States of America; 3 Department of Orthopaedics, University of Washington, Seattle, Washington, United States of America; University of Western Ontario, Canada

## Abstract

Emerging evidence suggests that connexin mediated gap junctional intercellular communication contributes to many aspects of bone biology including bone development, maintenance of bone homeostasis and responsiveness of bone cells to diverse extracellular signals. Deletion of connexin 43, the predominant gap junction protein in bone, is embryonic lethal making it challenging to examine the role of connexin 43 in bone in vivo. However, transgenic murine models in which only osteocytes and osteoblasts are deficient in connexin 43, and which are fully viable, have recently been developed. Unfortunately, the bone phenotype of different connexin 43 deficient models has been variable. To address this issue, we used an osteocalcin driven Cre-lox system to create osteoblast and osteocyte specific connexin 43 deficient mice. These mice displayed bone loss as a result of increased bone resorption and osteoclastogenesis. The mechanism underlying this increased osteoclastogenesis included increases in the osteocytic, but not osteoblastic, RANKL/OPG ratio. Previous in vitro studies suggest that connexin 43 deficient bone cells are less responsive to biomechanical signals. Interestingly, and in contrast to in vitro studies, we found that connexin 43 deficient mice displayed an enhanced anabolic response to mechanical load. Our results suggest that transient inhibition of connexin 43 expression and gap junctional intercellular communication may prove a potentially powerful means of enhancing the anabolic response of bone to mechanical loading.

## Introduction

Normal remodeling of bone requires synchronized activity between bone resorbing osteoclasts and bone forming osteoblasts, as well as the coordination of this process by osteocytes, by far the most abundant bone cell. An abundance of data generated over the last several years suggest that connexins and gap junctional intercellular communication (GJIC) play a critical role in coordinating synchronized bone cell activity, especially in response to extracellular signals including those induced by mechanical load [1], [2], [3]. Gap junctions are membrane spanning channels that allow passage of ions and signaling molecules, less than 1KD in size, between two adjacent cells. Each gap junction is composed of two hemi channels, or connexons, and each connexon is comprised of 6 connexins. The predominant connexin (Cx) in osteoblasts, osteoclasts and osteocytes is Cx43, but other connexins have been identified in bone cells [4], [5], [6], [7].

Most of the data supporting a role for Cx43 and GJIC in osteoblastic cell differentiation, coordinated cell responsiveness, and mechanotransduction comes from in vitro studies. For instance, studies from our laboratory [8], [9], [10] and others [11], [12], [13], [14] demonstrated that Cx43 expression and GJIC parallel osteoblastic differentiation and that inhibiting GJIC and Cx43 expression in many osteoblastic cell lines and primary cultures results in decreased expression of phenotypic characteristics of differentiated osteoblasts. Additionally, Schiller et al [15] showed that inhibiting GJIC induces the trans-differentiation of osteoblastic MC3T3-E1 cells and primary culture human osteoblastic cells to an adipocytic phenotype. In vivo studies demonstrated that global knock out of Cx43 in mice results in delayed skeletal ossification, craniofacial abnormalities, and osteoblast dysfunction [16]. Additionally, mutations in the human Cx43 gene lead to oculodentodigital dysplasia (ODDD) [17], [18], [19] characterized by, among other things, skeletal dysplasia.

While these studies strongly suggest that Cx43 and GJIC are critical to normal bone development and remodeling, they are limited by their in vitro nature or the fact that they reflect the consequence of global Cx43 deficiency during development only, as global Cx43 deletion is embryonic lethal [20]. To address these concerns, and examine the role of connexins in bone post development, murine models of Cx43 conditionally deleted in only osteoblasts and osteocytes have been developed [21], [22]. However, the bone phenotype described in these studies has been rather inconsistent, with some models displaying an osteopenic phenotype in some studies [23] but not others [24] and other models not displaying a basal bone phenotype at all [22].

To more closely examine this issue we selectively deleted the Cx43 gene in mouse osteoblasts and osteocytes using Cre recombinase driven by the human osteocalcin promoter, as previously reported by Plotkin et al [22], and evaluated the bone phenotype. We found that osteoblast and osteocyte specific ablation of Cx43 results in decreased bone mineral density (BMD) and bone strength due at least in part to increased osteoclastic activity. This increased osteoclastic activity is a result of a decrease in Cx43 and GJIC that results in alterations in the receptor activator of nuclear factor-κB-ligand)/osteoprotegerin (RANKL/OPG) pathway in osteocytes but not osteoblasts. Furthermore, in contrast to what previous in vitro studies predicted, mice with osteoblast and osteocyte specific deletion of Cx43 displayed an enhanced anabolic response to physiological mechanical load.

## Results

### Generation of Osteoblast and osteocyte specific Cx43 deficient mice

**Figure 1:**
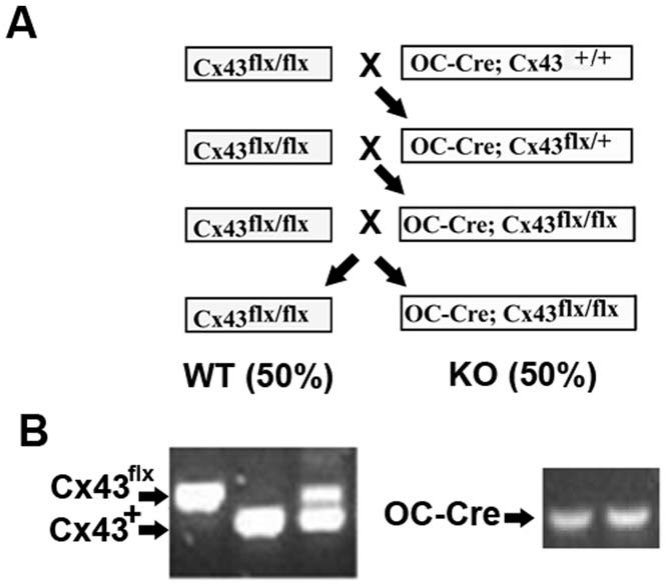
Breeding of Cx43 deficient mice. **A:** Breeding strategy for developing osteoblast and osteocyte Cx43 deficient mice and assessment of their genotype. **B:** Using the primers described in Table 1, genomic DNA extracted from ears was amplified for genotyping.

Using the protocol described in Materials and Methods (Figure 1A), we generated wild type equivalent (genotype: Cx43^flx/flx^, designated wild type), and conditional Cx43 deficient equivalent (genotype: OC-Cre; Cx43^flx/flx^, designated Cx43 deficient) mice.

The breeding strategy we used is more efficient for breeding Cx43 deficient mice than the method used by Plotkin et al [22]. This strategy was previously used to ablate type I IGF receptor in mouse osteoblasts and osteocytes [25]. These investigators estimated the rate of OC-Cre ablation of “floxed” sequences in osteoblasts and osteocytes to be approximately 88%.

**Figure 2:**
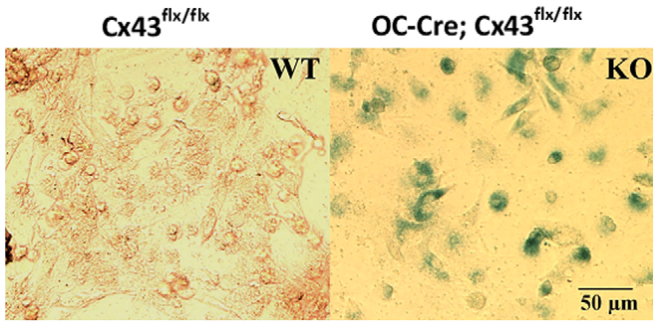
Confirmation of Cx43 deletion by X-gal staining. The figure shows X-gal staining of osteoblasts isolated from femurs and tibia of a wild type (Cx43^flx/flx^, WT) and a Cx43 deficient (OC-Cre;Cx43^flx/flx^, KO) mouse. Cells from wild type mice had no blue staining indicating that floxed Cx43 was not deleted in the absence of OC-Cre. Cells from a Cx43 deficient mouse were positive for β-galactodase indicating Cx43 deleted by Cre.

**Figure 3:**
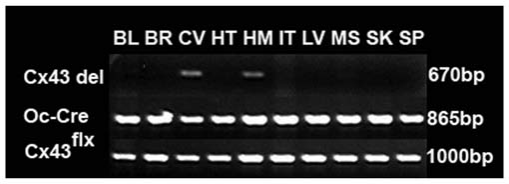
Confirmation of Cx43 deletion by PCR. The figure shows PCR of genomic DNA extracted from bladder (bld), brain (brn), calvaria (cal), heart (ht), humerus (hm), intestine (int), liver (lv), muscle (msl), skin (sk), and spleen (spl) from a OC-Cre; Cx43^flx/+^ mouse. DNA was amplified using primers (Cx43delForw and Cx43delRev) flanking the junction between the intron of Cx43 and LacZ region. If Oc-Cre is activated the floxed Cx43 is deleted allowing amplification of a 670 bp product. If Oc-Cre is not activated and floxed Cx43 is not deleted, the template is too large to be amplified. We observed a 670 bp PCR product in calvaria and humerus indicating Cx43 is deleted. The same DNA was amplified using primers for Cre and Cx43 were used as positive control.

Genotypes were determined by PCR as shown in Figure 1B using the primers described in Table 1. A LacZ reporter gene was inserted after Cx43^flx/flx^, and deletion of Cx43 results in expression of β-galactosidase [21]. Therefore, we examined X-gal staining in osteoblastic cells isolated from femurs. Osteoblastic cells from Cx43 deficient (Oc-Cre;Cx43^flx/flx^) mice displayed positive X-gal staining but osteoblastic cells from wild type (Cx43^flx/flx^) mice did not (Figure 2), demonstrating that Oc-cre expression resulted in efficient deletion of the ‘floxed’ Cx43 alleles. To determine the specificity of Cx43^flx^ deletion, we used primers (DelForw and DelRev in Table 1) flanking the junction between the intron of the Cx43 and LacZ coding region from the deleted allele to amplify genomic DNA isolated from bones as well as other tissues. A 670 bp amplicon was obtained from DNA isolated from whole femurs and calvaria of an Oc-Cre; Cx43^flx/flx^ mouse but was absent from DNA extracted from soft tissues from the same mouse (Figure 3). We also used the same DNA to amplify OC-Cre and Cx43 as a control and detected an 865 bp Cre band and a 1KB Cx43^flx^ band in all tissues examined (Figure 3). These data (Figures 2 and 3) indicate that “floxed” Cx43 alleles were deleted by OC-Cre in both osteoblasts and osteocytes.

**Table 1:** Primers used for genotyping.

Primer	Sequence 5′→3′	Allele	Length
Cre1	TGA TGG ACA TGT TCA GGG ATC	Cre	865 bp
Cre2	CAG CCA CCA GCT TGC ATG A		
UMP	TCA TGC CCG GCA CAA GTG AGA C	Cx43^flx^ and Cx43^+^	1 KB and 900 bp
UMPR	TCA CCC CAA GCT GAC TCA ACCG′		
43delforw	GGC ATA CAG ACC CTT GGA CTC C	Tissue specific deletion	670 bp
43delrev	TGC GGG CCT CTT CGC TAT TAC G		

### Deletion of Cx43 reduced GJIC in osteoblasts

**Figure 4:**
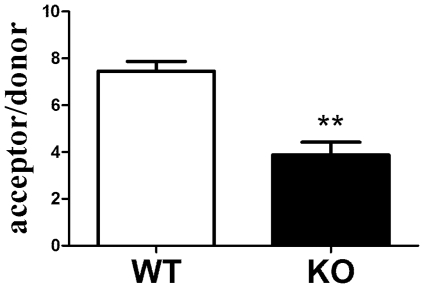
Quantification of GJIC in osteoblasts isolated from wild type (WT) and Cx43 deficient (KO) mice. Donor hFOB cells were placed in contact with a monolayer of acceptor cells isolated from femurs from wild type or Cx43 deficient mice. Bars represent the mean±SD number of acceptor cells to which dye transferred from each donor hFOB cells. n = 4 **p<0.01 vs wild type.

To determine whether deletion of Cx43 decreased GJIC in bone cells, we isolated osteoblastic cells from femurs and tibias and completed dual label dye transfer assays. GJIC was decreased approximately 50% in osteoblastic cells isolated from Cx43 deficient mice relative to those isolated from wild type mice (Figure 4). Cx45 and Cx26 mRNA levels were similar in wild type and Cx43 deficient mice (data not shown). These data confirm that the majority of GJIC communication in osteoblasts, as assessed by dye transfer, is mediated by gap junctions composed of Cx43.

### Cx43 conditional deficient mice display decreased BMD and have weaker bones

**Figure 5:**
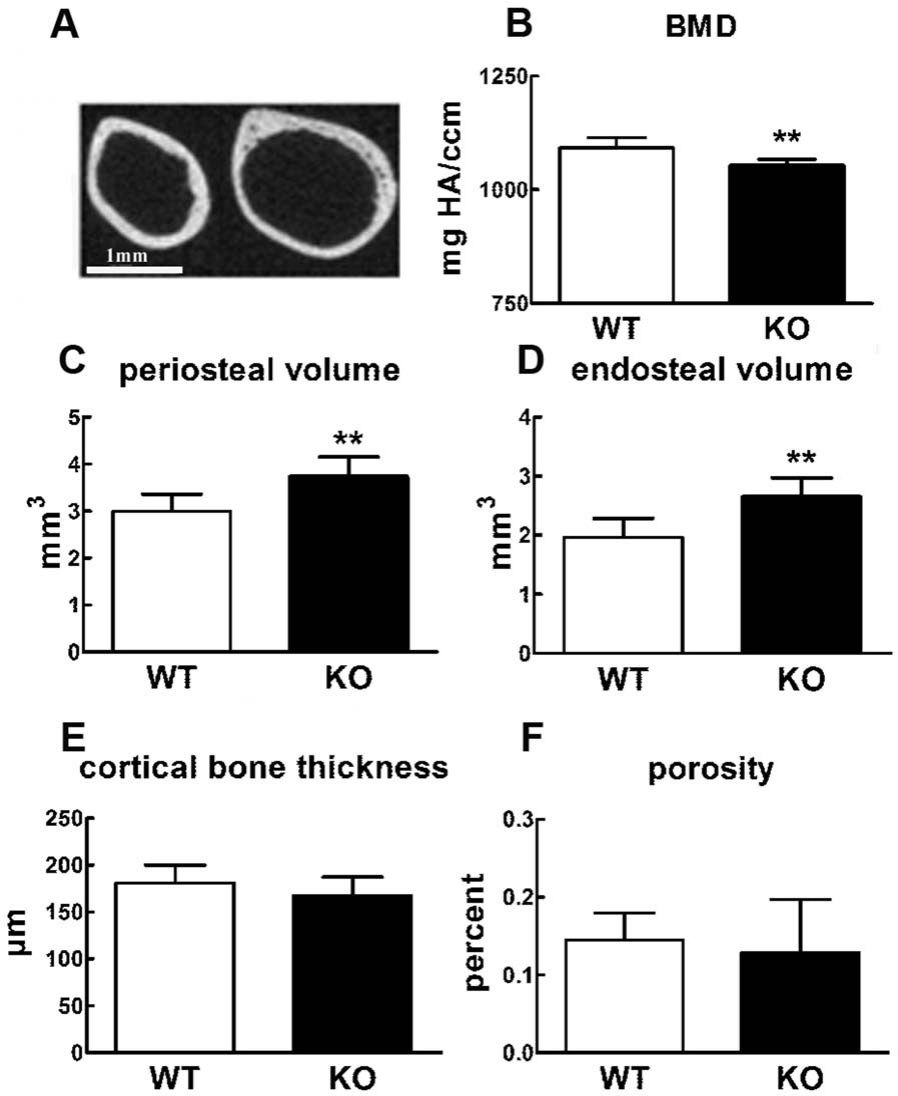
Microstructure of the femur midshaft of wild type (WT) and Cx43 deficient mice (KO). μCT image of femoral midshaft of 8 week old wild type (**A left**) and Cx43 deficient mice (**A right**) mice. Periosteal and endocortical expansion is evident in Cx43 deficient mice relative to wild type. **B–F:** Quantification of μCT in 8 week old Cx43 deficient mice and wild type mice. Cx43 deficient mice displayed a significant decrease in BMD (**B**) and increase in periosteal (**C**) and endocortical volumes (**D**). Cx43 deficient mice displayed no change in cortical bone thickness (**E**, p = 0.054) or bone porosity (**F**, p = 0.55). n = 9 mice per group, ** p<0.01, Bar = 1 mm.

To examine the bone phenotype of Cx43 deficient and wild type mice we examined 8 week and 6 month old male mice using micro-computed tomography (μCT). μCT analysis of the midshaft of the femur revealed periosteal and endosteal expansion in Cx43 deficient mice relative to wild type mice (Figure 5A). The femur midshafts of 8 week old Cx43 deficient mice displayed statistically significant 3.6% reductions in BMD (Figure 5B) and 24.7% and 35.4% increases in peri- and endosteal volume, respectively, relative to wild type mice (Figure 5C and D). However, the cortical bone thickness (Figure 5E) and porosity (Figure 5F) were not significantly different. Femur midshafts from 6 month old Cx43 deficient mice also displayed significantly reduced BMD (4.1%) and increased periosteal (29.6%) and endosteal volume (46.0%) relative to wild type mice. However, cortical bone thickness and porosity were not significantly different.

**Figure 6:**
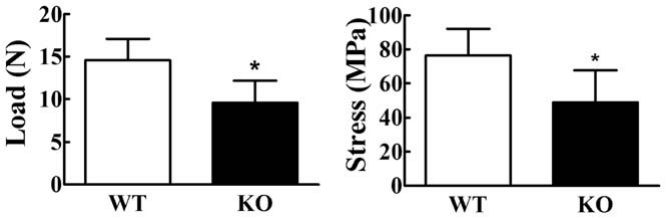
Strength of femurs from 8 week old mice. Femurs were loaded to failure using 3-point bending and maximum tensile stress at failure was determined form force deformation curves. Both load (**left**) and stress (**right**) at failure were significantly lower in Cx43 deficient mice (KO) relative to wild type (WT) mice. n = 6–7 mice per group, *p<0.05 vs wild type.

Femurs from 8 week old Cx43 deficient mice displayed a 35% and 36% decrease in whole bone strength and material strength, respectively, compared to wild type mice (Figure 6).

### Cx43 deficiency increases bone formation rate

**Figure 7:**
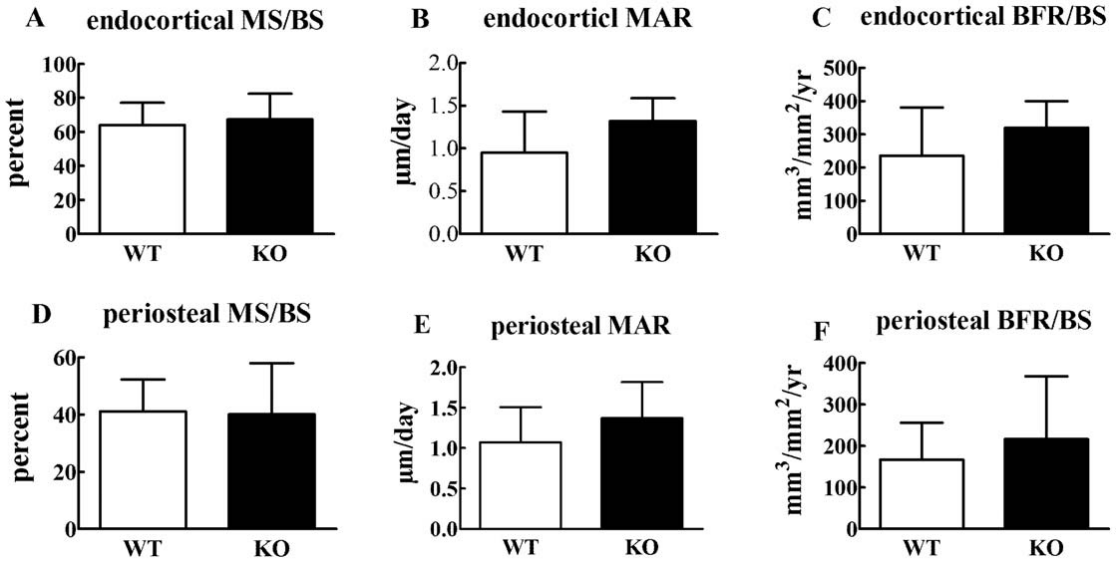
Dynamic histomorphometry in 8 week old mice. Eight week old Cx43 deficient (KO) and wild type (WT) mice displayed similar endocortical or periosteal mineralized surface (MS/BS, **A, D**) bone mineral apposition rates (MAR, **B, E**) and bone formation rates (BFR/BS, **C, F**). n = 6 mice per group.

**Figure 8:**
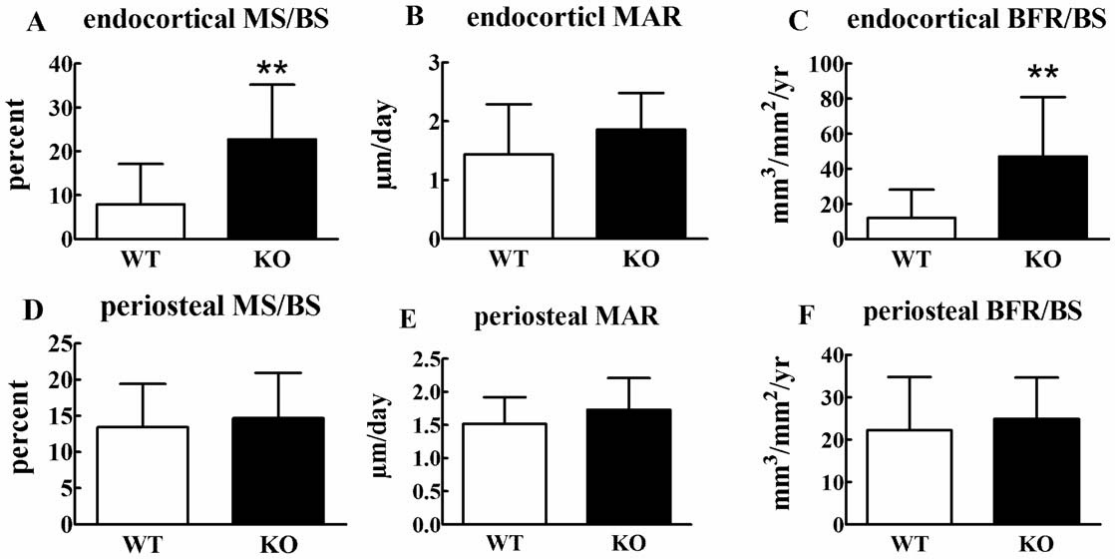
Dynamic histomorphometry in 6 month old mice. Six month old Cx43 deficient (KO) mice show significantly increased endocortical MS/BS (**A**), and BFR (**C**), but no significant change in other parameters (B, D, E and F). n = 4–5 mice per group, ** p<0.01.

**Figure 9:**
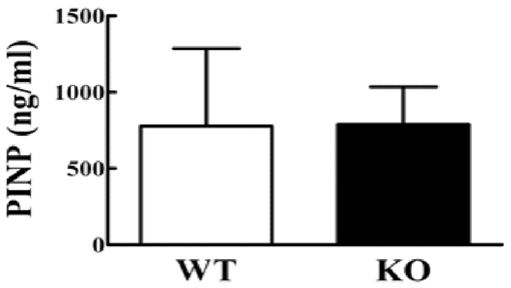
Quantification of serum markers of bone formation. Four week old Cx43 deficient (KO) and wild type (WT) control mice have similar PINP concentrations in serum. n = 3–9 mice per group.

Since bone mass is balanced by bone formation and resorption, we next examined, using dynamic histomorphometry and quantification of a serum bone formation marker, whether the phenotype in Cx43 deficient mice is due to decreased bone formation. Eight week (Figure 7) and 6 month (Figure 8) old mice ulna endocortical and periosteal bone mineral surface (MS/BS), bone mineral apposition rate (MAR), and bone formation rate (BFR/BS) were quantified. These three parameters were similar in 8 week old Cx43 deficient and wild type mice ulnae both at the endosteal and periosteal surfaces. (Figure 7). However, significant increases in endosteal MS/BS and BFR/BS, but not MAR, were observed in 6 month old Cx43 deficient ulnae (Figures 8A, B and C). No significant differences were detected in periosteal surfaces of 6 month old mice (Figure 8D, E and F). We also found that serum levels of the bone formation marker, procollagen I amino-terminal propeptides (PINP) in the serum of 4 week old Cx43 deficient and wild type mice were similar (Figure 9). Taken together these data suggest that bone formation was increased in Cx43 deficient mice, relative to wild type mice, but this difference did not reach statistical significance until the mice were older, i.e. 6 months.

### Cx43 deficiency results in increased bone resorption and osteoclastogenesis

**Figure 10:**
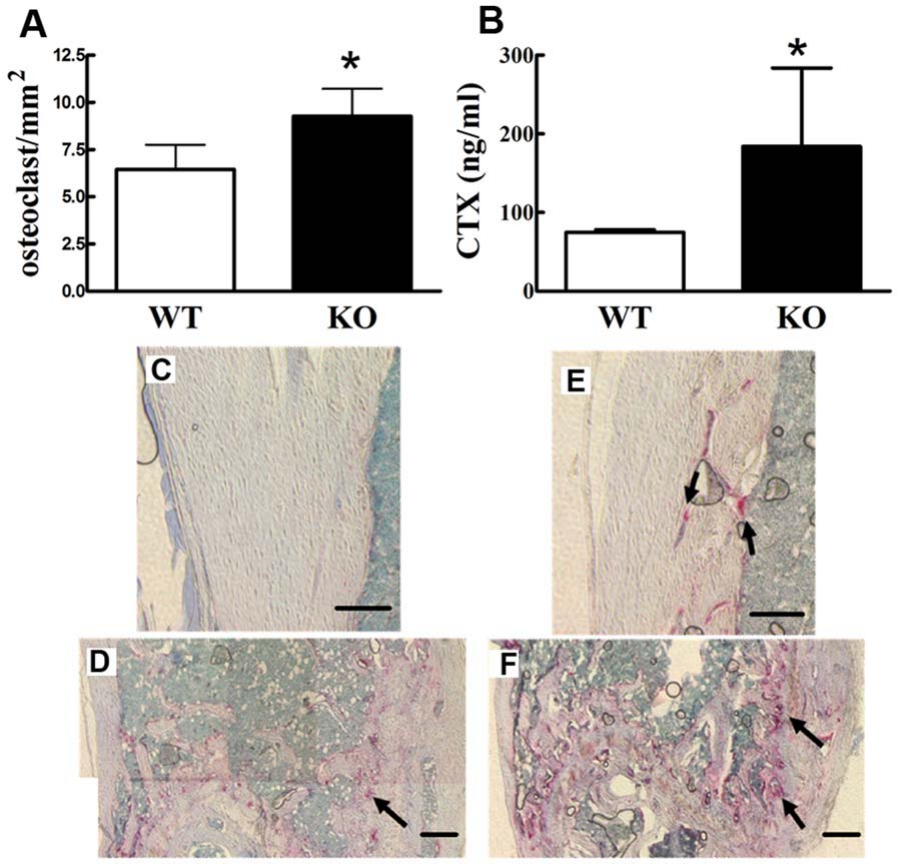
Cx43 deficient mice display increased bone resorption and osteoclast number. Four week old Cx43 deficient mice show increased osteoclast number in the distal epiphyseal area (**A**) and increased serum CTX concentration (**B**). n = 4 (**A**) or 3–9 (**B**) mice per group, * p<0.05. **C–E:** Typical TRAP staining of four-week old wild type (WT) (**C** and **D**) and Cx43 deficient (KO) (**E** and **F**) mouse femur. There are more osteoclasts in bone from Cx43 deficient mice. Scale bar = 100 µm.

Cx43 deficient mice displayed more TRAP positive cells than did wild type mice in femur cortical and trabecular areas (Figures 10A–D). We quantified osteoclast number in epiphyseal trabecular bone of the femur and found that Cx43 deficient mice displayed statistically significant increases in osteoclast number compared to wild type (Figure 10E). Furthermore, serum Type I collagen carboxyl-terminal crosslinking telopeptide (CTX) concentration, which is a product of collagen breakdown during osteoclastic bone resorption, serum levels of which reflect degree of bone resorption [26], increased approximately 1.5 fold in Cx43 deficient mice relative to wild type mice (Figure 10F). Taken together these results suggest increased bone resorption and osteoclastogenesis in Cx43 deficient mice.

### Cx43 in osteocytic but not osteoblastic cells regulates the RANKL and OPG mRNA level ratio

**Figure 11:**
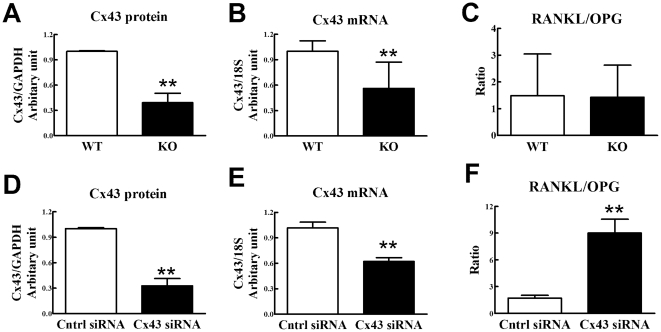
Ablation of Cx43 affects RANKL/OPG mRNA ratio in osteocytic but not osteoblastic cells. Cx43 protein (**A**) and mRNA (**B**) levels in primary osteoblasts from Cx43 deficient (KO) mice were decreased relative to wild type (WT), but the ratio of RANKL/OPG mRNA levels were similar in osteoblasts from Cx43 deficient and wild type mice (**C**). Osteocytic MLO-Y4 cells were transfected with either scrambled control siRNA or siRNA directed against Cx43 mRNA. MLO-Y4 cells transfected with Cx43 siRNA displayed decreased Cx43 protein (**D**) and mRNA levels (**E**) but increased RANKL/OPG mRNA levels (**F**), relative to cells transfected with control siRNA. n = 3 separate experiments for **A** and **D**; 11–12 for **B** and **C**; and 10 for **E** and **F**), ** p<0.01.

The RANKL-OPG axis is a major pathway through which osteoblasts and osteocytes regulate osteoclastic bone resorption. To examine the mechanism by which Cx43 deficiency in osteoblasts and osteocytes leads to increased osteoclastogenesis and bone resorption, we quantified the ratio of RANKL mRNA levels to OPG mRNA levels (RANKL/OPG ratio), using real time RT-PCR, in primary osteoblastic cells from Cx43 deficient and wild type mouse long bones. Steady state Cx43 protein and mRNA levels in osteoblastic cells from Cx43 deficient mice were significantly decreased relative to levels in osteoblastic cells from wild type mice, confirming Cx43 deficiency in Cx43 deficient mice (Figures 11A and B). However, the RANKL/OPG mRNA ratio was not significantly different in osteoblastic cells from Cx43 deficient and wild type mice (Figure 11C), which suggests that ablation of Cx43 in osteoblasts does not affect osteoclastogenesis through the RANKL-OPG pathway.

We next examined the RANKL/OPG mRNA ratio in osteocytic cells. Because it is challenging to isolate homogeneous populations of primary osteocytic cells, making interpretation of results difficult, we used MLO-Y4 as model osteocytic cells. MLO-Y4 cells were rendered Cx43 deficient by transfection with siRNA directed against Cx43. As controls we examined MLO-Y4 cells transfected with scrambled siRNA. We previously demonstrated that MLO-Y4 cells transfected with Cx43 siRNA, identical to the cells we used in this study, display significantly lower Cx43 protein levels compared to MLO-Y4 cells transfected with scrambled siRNA [59]. Transfection of Cx43 siRNA significantly reduced Cx43 protein and mRNA levels in MLO-Y4 cells (Figure 11D and E). Interestingly, Cx43 deficient MLOY-4 cells displayed a fivefold increase in the RANKL/OPG mRNA ratio relative to scramble control MLO-Y4 cells (Figure 10D). These data suggest that Cx43 levels in osteocytes, but not osteoblasts, increase osteoclastogenesis via altering the RANKL/OPG mRNA ratio.

### Ablation of Cx43 results in an enhanced response to physiologic magnitude mechanical loading

**Figure 12:**
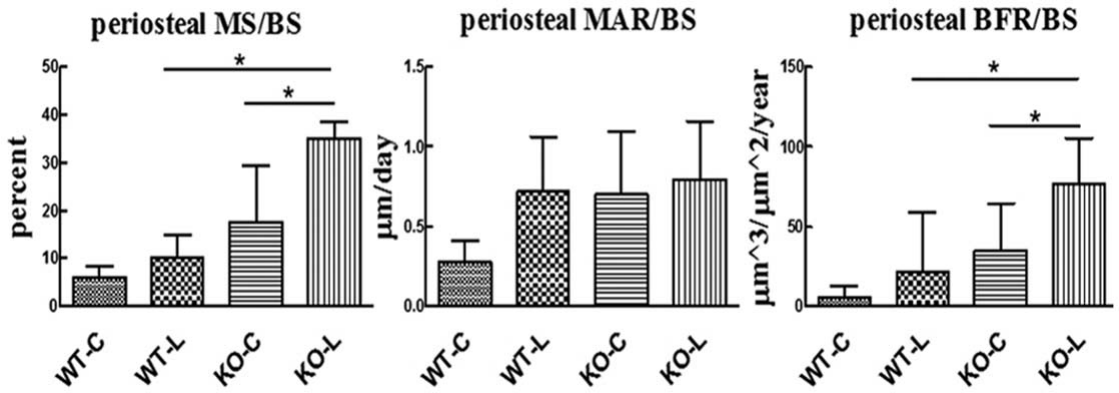
Mechanical loading of bone in Cx43 deficient and wild type mice. An enhanced periosteal osteoblastic response was observed in 6 month old Cx43 deficient mice (KO) relative to wild type (WT) when the right tibias of each were exposed to mechanical loading that induced equivalent peak normal strains. p.MS, p.MAR and p.BFR are reported for contralateral (C) and loaded tibias (L). p.MS and p.BFR were significantly greater in loaded tibia, relative to contralateral, in Cx43 deficient mice but not wild type mice. p.Mar in loaded tibia was not significantly different than in contralateral tibia in Cx43 deficient or wild type mice. n = 7–9 mice per group, * p<0.05.

At 6 months of age, tibiae of Cx43 deficient mice dispalyed p.MS, p.MAR, and p.BFR values that were not statistically significantly different than wild type mice (Figure 12). In wild type mice, the mechanical loading regimen did not induce significant increases in p.MS, p.MAR, or p.BFR compared to contralateral tibiae. In contrast, mechanically loaded tibiae in Cx43 deficient mice demonstrated significantly increased p.MS (132%, p = 0.003), and p.BFR (217%, p = 0.001) compared with contralateral tibiae (p.MAR was not significantly elevated). The 151% greater p.BFR in the loaded tibiae of Cx43 deficient mice vs loaded tibiae of wild type mice was primarily achieved via 222% greater p.MS.

## Discussion

Emerging data suggest that Cx43 mediated GJIC is critical to normal bone development. Unfortunately, examination of the role of Cx43 post development is challenging because mice with global Cx43 deficiency die at birth [16], [20]. However, using a Collagen I promoter controlled Cre and “floxed” Cx43 system, Chung et al [23] reported that conditional ablation of Cx43 in only osteoblasts and osteocytes resulted in decreased whole body BMD, as determined by DEXA, decreased trabecular BV/TV, as determined by dynamic histomorphometry, and decreased osteoblastic number, with no accompanying change in osteoclastic number. Interestingly, in a subsequent study this group reported that the same Cx43 deficient model displayed cortical thinning and increased marrow size, relative to wild type, but no change in site specific BMD or histomorphometric parameters [24]. Further complicating the issue, Plotkin et al [22] reported that using an osteocalcin promoter controlled Cre and floxed Cx43 system to ablate Cx43 in osteoblasts and osteocytes, albeit at a later stage in osteoblastic differentiation, did not result in changes in site specific BMD as quantified by DEXA [22] None of the aforementioned studies examined biomechanical strength of bone in Cx43 deficient mice. Thus, the role of connexins and GJIC in adult bone metabolism remains unresolved.

To further address this issue we deleted Cx43 in osteoblasts and osteocytes using the osteocalcin driven Cre and floxed approach taken by Plotkin et al [22]. We demonstrated that deletion of Cx43 in bone cells significantly reduced GJIC confirming that Cx43 is the predominant connexin in bone. However, unlike the studies of Plotkin et al, our data showed that specific deletion of Cx43 in osteoblasts/osteocytes leads to a phenotype that includes decreased BMD and, importantly, decreased mechanical strength (Figures 5 and 6). Furthermore, whereas Chung et al and Grimston et al did not detect a significant change in histomorphometric parameters we detected a moderate increase in both endosteal and periosteal MAR and BFR/BS in 8 week old Cx43 deficient mice ulnae (Figure 7) and a statistically significant increase in MAR and BFR/BS in 6 month old Cx43 deficient mouse ulnae relative to wild type control mice (Figure 8).

One possible explanation as to why our results differ from those previously reported is that we used μCT and dynamic histomorphometry to access overall adult bone morphology, as well as bone formation and resorption, whereas previous studies used DEXA alone or DEXA combined with dynamic histomorphometry, which may be less sensitive approaches than μCT. Furthermore, in the case of the murine model developed by Chung et al [23] and Grimston et al [24], Cx43 deficiency was driven by the Collagen 1 promoter and therefore occurred at an earlier point in osteoblastic differentiation relative to the model we used, wherein the Cx43 deficiency is driven by the osteocalcin promoter and therefore occurs later in osteoblastic differentiation. This difference could account for some of the differences in phenotype between the models. In any case, our finding of decreased mechanical strength and increased markers of osteoclastic bone resorption in Cx43 deficient mice, relative to wild type, when combined with our μCT and histomorphometric data, strongly suggest an osteopenic phenotype in mice with Cx43 deficient osteoblasts and osteocytes.

Since deleting Cx43 in osteoblasts and osteocytes did not affect mouse ambulation (data not shown), our data indicate that functional Cx43 in osteoblasts and osteocytes is required for normal bone mass acquisition and maintenance. Our finding that MAR and BFR are not reduced, and if anything are increased, in Cx43 deficient mice, together with those of Plotkin, Chung and Grimston showing no, or only slight, increases in MAR and BFR in a similar mouse model, suggests that a decrease in osteoblastic activity does not contribute to the bone phenotype we observed in osteoblast and osteocyte specific Cx43 deficient mice.

Because our data suggest that decreased osteoblastic function was not mediating the cortical bone phenotype in Cx43 deficient mice we examined another possible explanation, increased osteoclastic bone resorption. Interestingly, TRAP staining revealed that Cx43 deficient mice have more osteoclasts than wild type mice (Figure 10A–E). Cx43 deficient mice also displayed increased serum levels of the bone resorption marker CTX (Figure 10F), but no significant change in levels of the bone formation marker PINP, relative to wild type mice (Figure 9). These data suggest that ablation of Cx43 in mouse bone cells leads to decreased BMD and weakened bones due to increased bone resorption.

We next sought to determine how Cx43 deficiency could result in increased osteoclast differentiation or activity. A major regulator of osteoclast formation and activity is the RANKL/OPG system. RANKL, a ligand expressed on the surface of marrow stromal cells and osteoblastic precursors, binds to its receptor found on the surface of osteoclastic precursor cells and thereby activates osteoclastogenesis. OPG serves as a soluble decoy molecule and binds with RANKL thus blocking its activity and inhibiting osteoclastogenesis. The ratio of RANKL to OPG, therefore, provides a reliable indicator of relative osteoclastogenesis. However, we found the RANKL/OPG mRNA ratio in osteoblastic cells isolated from Cx43 deficient mice was similar to that in osteoblastic cells isolated from wild type mice. Therefore, it is unlikely that Cx43 regulation of the RANKL/OPG pathway in osteoblastic cells contributed to altered osteoclastogenesis.

We next examined whether Cx43 deficiency in osteocytic cells could alter the RANKL/OPG ratio and thereby contribute to decreased osteoclastogenesis. Osteocytes are the most abundant cells in bone [27] and are well coupled with each other and osteoblasts via GJIC [28], [29], [30] and GJ are the key couplers in the osteocyte syncytium [31], [32]. Osteocytes regulate bone formation by mediating, via GJIC, the effects of mechanical load on osteoblastic differentiation [33]; through the release of osteoblast activating factors including nitric oxide [34] and prostaglandins [35]; and by modulation of Wnt/β-catenin signaling [36]. Osteocytes can also regulate bone resorption [37]. Targeted ablation of osteocytes in vivo results in increased RANKL/OPG expression ratios, increased osteoclast number and osteopenia [38]. Furthermore, osteocyte-like MLO-Y4 cells express RANKL and induce osteoclast formation and activation [39] and You et al showed that exposure to fluid flow inhibits MLO-Y4 induced osteoclastic bone resorption via decreasing the RANKL/OPG ratio [40]. Interestingly, we have previously demonstrated that fluid flow similar to that examined by You et al [40] also increases Cx43 expression and phosphorylation [41], indirectly linking Cx43 activity with the RANKL/OPG pathway. Taken together these data suggest that Cx43 deficiency may affect the osteocytic RANKL/OPG ratio in osteocytes. In strong support of this concept, using MLO-Y4 osteocytic cells as a model, our data demonstrate that Cx43 deficient osteocytic cells display an increased RANKL/OPG mRNA ratio (Figure 11F) which may explain the increased osteoclastogenesis and increased bone resorption we observed in Cx43 deficient mice. This is the first direct evidence of a link between connexins and the RANKL/OPG pathway. It is unlikely that the regulation by Cx43 and GJs of the RANKL/OPG pathway is related to the heterotypic GJIC between osteocytic MLO-Y4 cells and osteoclastic cells as it occurred in MLO-Y4 in mono culture in the absence of osteoclasts. However, this does not preclude a role for homotypic GJIC between MLO-Y4 cells in the regulation of the RANKL/OPG pathway.

The mechanism by which Cx43 and GJIC interacts with the RANKL/OPG pathway is unclear. However, published reports suggest that the activity of SP-1/SP-3 transcription factors is regulated by Cx43 mediated GJIC in osteoblastic cells [42], [43] and SP1 an SP3 are implicated in the regulation of both RANKL and OPG gene expression [44], [45], [46]. Therefore, Cx43 and GJIC regulation of RANKL and OPG could be mediated by effects on SP1, SP3, or both.

Emerging data suggest that Cx43 and GJIC contribute to mechanotransduction in bone [41], [47]. For instance, cyclic stretch has been shown to increase Cx43 protein expression and GJIC in osteoblastic cells *in vitro* [47], and we [41] and others [1], [48], [49] demonstrated that fluid flow increases GJ expression and function in osteocytic MLO-Y4 cells. Additionally, GJs communicate mechanical signals detected by osteocytic MLO-Y4 cells to osteoblastic cells thereby increasing osteoblastic differentiation [33]. *In vivo*, Lozupone et al [50] demonstrated that mechanical loading of rat metatarsal bones increased the incidence of osteocytic GJs and we demonstrated that expression of Cx43 by osteocytes is increased in areas of bone exposed to tension relative to areas exposed to compression or to control bone [51]. Interestingly, we also demonstrated that GJIC deficient bone cells are dramatically less responsive to parathyroid hormone [10], fluid flow [2], [52] and electric fields [53] than are normal osteoblastic cells. Thus, in vitro GJIC contributes to the responsiveness of osteoblastic cells to diverse extracellular signals, including those that are believed to mediate the anabolic effects of mechanical load. In this context one might predict that bone with Cx43 deficient osteoblasts and osteocytes would be less responsive to the anabolic effects of mechanical load. Indeed, using a Collagen I promoter controlled Cre and “floxed” Cx43 deficient mouse model and three point bending, Grimston et al reported that increased endocortical BFR and MAR in response to mechanical load was attenuated in osteoblast and osteocyte specific Cx43 deficient, relative to wild type, mice [24].

In contrast, our in vivo mechanical loading data clearly demonstrate that deletion of Cx43 in osteoblasts and osteocytes results in an enhanced periosteal response to physiological mechanical loading. That these data contrast with those of Grimston et al may be due to differences in basal levels of periosteal osteoblast function, magnitude of induced strains (low level osteogenic stimulus in the current study versus a stimulus sufficient to induce periosteal woven bone formation in the study by Grimston et al [24]) and differences in mechanotransduction at the periosteal versus endocortical surface. Furthermore, our loading studies were completed on 6 month old mice, a period at which peak bone mass was attained, while the studies by Grimston et al [24], were completed on 4 month old mice. In any case, we believe our data are internally consistent with the cortical bone phenotype of Cx43 deficient mice (i.e., an expanded periosteal envelope) which reflects an accumulation of elevated osteoblast activity over time. We do not know whether the enhanced response to loading was achieved at the level of the osteocyte or osteoblast (or both).

In their entirety, we believe these data provocatively suggest that gap junctions composed of Cx43 may serve, in vivo, as conduits for intercellular signaling that inhibits osteoblastic activity at the periosteal surface and osteoclastic activity at the endocortical surface. Thus, eliminating Cx43 and GJIC results in increased osteoblastic and osteoclastic activity, as evidenced by accelerated cortical expansion. While the mechanism of increased osteoblastic activity is unclear the increased osteoclastic activity is a result of an increase in the RANKL/OPG ratio. Inhibition of Cx43 expression would then result in both increased osteoblastic bone formation and an even greater unbalanced increase in osteoclastic bone resorption, resulting in a phenotype characterized by decreased BMD and weaker bones. Exposing Cx43 deficient mice to mechanical load may decrease the osteocytic RANKL/OPG ratio, as suggested by in vitro studies [40], thus decreasing osteoclastic activity, in the context of increased osteoblastic activity. This would explain the increased anabolic response to mechanical load in Cx43 deficient mice. Additionally or alternatively, the increased osteoblastic activity at the periosteal surface may account for the increased anabolic response to mechanical load.

In summary, our data suggest that deletion of Cx43 in osteoblasts and osteocytes leads to decreased BMD and weaker bones. In vitro studies suggest that this phenotype may, at least partly, be a result of an incrased RANKL/OPG ratio in osteocytes, but not osteoblasts, resulting in increased osteoclast formation. Importantly, Cx43 deficient mice display an enhanced bone anabolic response to mechanical loading. Thus, transient inhibition of connexin expression and GJIC may prove a potentially powerful means of enhancing bone's anabolic response to mechanical loading.

## Materials and Methods

### Transgenic mice

Mice expressing Cre recombinase under the control of the human osteocalcin promoter (abbreviated as OC-Cre; Cx43^+/+^) [25] were first bred with mice in which the Cx43 gene is flanked by two loxP sites (Cx43^flx/flx^) [21] to generate OC-Cre; Cx43^flx/+^ mice. Next, we crossed OC-Cre; Cx43^flx/+^ with Cx43^flx/flx^ to generate OC-Cre; Cx43^flx/flx^. We then back bred OC-Cre; Cx43^flx/flx^ mice with Cx43^flx/flx^ mice to generate equal number of OC-Cre; Cx43^flx/flx^ (Cx43 deficient equivalent) and Cx43^flx/flx^ (wild type) littermate mice. Mice derived from this breeding strategy were bred with C57BL/6 for 3 generations resulting in mice with a C57BL/6 background. Genotyping was performed by PCR using genomic DNA isolated from mouse ear pieces and primers listed in Table 1. To test the specificity of Cx43 ablation, DNA was isolated from bladder, brain, calvaria, heart, humerus, intestine, liver, muscle, skin, and spleen using a Qiagen DNeasy Tissue Kit (Qiagen) and amplified with Cx43delForw and Cx43delRev primers (Table 1). Soft tissue and marrow were removed before DNA was isolated from calvaria and humerus. The DNA from calvaria and humerus should contain DNA from both osteoblasts and osteocytes. All animal work was conducted according to relevant national and international guidelines and was approved under Penn State College of Medicine's IACUC protocol #94-120.

### Cell culture

Two cell lines were used in this study. The immortalized osteocytic cell line MLO-Y4, (kindly provided by Dr. Lynda Bonewald), was cultured on type I collagen (BD Laboratory) coated plates in Modified Eagle Medium Alpha (αMEM, GIBCO) supplemented with 5% fetal bovine serum (FBS), 5% calf serum (CS), and 1% penicillin and streptomycin (P/S). The human fetal osteoblastic progenitor cell line hFOB 1.19 cells (kindly provided by Dr. Steven Harris) was maintained using Dulbecco's modified Eagle's medium (DMEM)–Ham's F-12 1∶1 media (GIBCO) supplemented with 10% FBS and 1% P/S.

Primary osteoblastic cells were isolated from explant cultures of 3 week old Cx43 deficient or wild type femurs and tibias which were digested in 1 mg/mL collagenase (Sigma, St Louis, MO) for 75–120 min at 37°C in a shaking water bath. The bones were cut into small pieces and the pieces from one femur and one tibia placed into one well of a 6 well plate in 600 µL of αMEM, with 10% FBS, 1%PS, 1% antimycotic (Sigma, St Louis, MO), 50 µg/mL ascorbic acid phosphate (Wako, Richmond, VA) for 5 days. The media was replaced with fresh media without antibiotics for one week. Cells that grew out from the bone fragments were then sub-cultured into 100 mm dishes. When the cells were 90% confluent gap junctional communication, protein and mRNA levels were quantified.

### Quantification of gap junctional intercellular communication

GJIC was quantified using a dual label fluorescent dye transfer technique and flow cytometry as previously described [54]. Donor hFOB cells were labeled with a fluorescent dye mixture containing 20 µl of calcein AM and 7 µl of 1,1′-dioctadecyl-3,3,3′,3′-tetramethylindolcarbocyanine perchlorate (DiI) (Molecular Probes, Eugene, OR) in 2 mL of bovine serum albumin-enriched phosphate buffered saline (PBS) with 20 µl of pluronic acid (Molecular Probes). Five thousand hFOB donor cells were dropped into one well of a 6 well plate covered with a monolayer of unlabeled acceptor osteoblasts from femurs and tibias of wild type or Cx43 deficient mice and incubated for 90 min at 37°C. Cells were released with trypsin and analyzed via fluorescence-activated cell sorter analysis (FACS; FACScan; BD-Pharmingen) as previously described [33]. Briefly, transfer of cytosolic calcein from hFOB donors to murine acceptor osteoblastic cells was quantified by analyzing the number of cells falling within the “acceptor” gate. The acceptor gate was determined using fluorescence intensity and set to exclude 99% of negative cells and 99.25% of donors.

### μCT to assess bone microstructure

Femurs from the right side of 8 week and 6 month old mice were harvested for μCT analysis. The diaphyses were scanned starting at the midpoint of the bone and acquiring 76 slices distally using a Scanco vivaCT 40 (Scanco Medical AG, Brüttisellen Switzerland) with scan settings of 55 KVp, 145 µA, and 200 ms integration time. Images were reconstructed as a matrix of 2048×2048×76 isotropic voxels measuring 10.5 µm. Images were gaussian filtered (sigma = 0.8, support = 1) and a threshold (24% of full scale) was applied to remove the surrounding soft tissue. The periosteal and endosteal boundaries of the cortical bone were segmented using the Scanco semi-automated edge detection algorithm. Periosteal volume, endosteal volume, bone porosity, cortical bone thickness, and BMD were calculated for the diaphysis of each femur using the Scanco Image Processing Library routines.

### Bone mechanical testing

Femurs from 8 week old mice were stored in PBS at −80°C before being mechanically tested to failure in three-point bending using an MTS MiniBionix 858 testing apparatus (MTS Systems, Eden Prairie, MN, USA) [55]. The flexural support spans were 8 mm while a loading rate of 1 mm/minute was applied. Femurs were consistently oriented so that loading occurred in the medial to lateral direction. All testing was executed with the bones hydrated and at ambient temperature.

### Material property calculation

Using the midshaft μCT image to calculate the moment of inertia, the ultimate tensile stress was calculated using Euler-Bernoulli beam theory. The ultimate tensile stress was calculated using the flexural formula (stress = FLc/4I), where F is the ultimate load, L is the support span length, c is the perpendicular distance from the neutral axis to the most lateral point on the bone surface, and I is the bending moment of inertia calculated about the neutral axis.

### Dynamic histomorphometry

For examination of 8 week and 6 month old mice, male mice were given an intraperitoneal injection of calcein (Sigma) at 10 mg/kg body weight and 6 days later injected with Alizarin red (Sigma) at 30 mg/kg body weight. Seven days after the second injection mice were sacrificed and ulnae were dissected and embedded using an Osteo-Bed bone embedding kit (Polysciences, Warrington, PA) following the manufacturer's protocol. The embedded ulnae midshafts were then cut into approximately 100 µm thick discs followed by abrasive polishing to a thickness of about 50 µm with lapping films (3 M, St. Paul, MN). Digital images were obtained using a confocal microscope (Leica, Bannockburn, IL). The following periosteal and endcortical bone parameters were quantified with ImageJ (NIH, Bethesda, MD): total perimeter (B.Pm); single label perimeter (sL.Pm); double label perimeter (dL.Pm), and double label area (dL.Ar). The following values were then calculated: mineralizing surface (MS/BS = [1/2sL.Pm+dL.Pm]/B.Pm×100; %); mineral apposition rate (MAR = dL.Ar/dL.Pm/6 days; µm/day) and bone formation rate (BFR/BS = MAR×MS/BS×3.65; µm^3^/µm^2^ per year) [56].

### Quantification of mouse serum bone formation and resorption markers

Serum was collected from 4 week old mice fasted for 8 hours. Type I collagen N-terminal propeptide (PINP) and C-terminal telopeptides (CTX) were measured with EIA or ELISA kits from Immunodiagnostic Systems Inc (IDS, Fountain Hills, AZ) according to manufacturer's protocols.

### Histological identification of osteoclasts

Right femurs from 6 month old mice were fixed in 4% paraformaldehyde for 3 days prior to decalcification with 10% EDTA (pH 7.4) for 5 days. These specimens were embedded in paraffin and cut into 5 µm sections then subjected to TRAP staining using a technique modified from Erlebacher and Derynck [57]. Briefly, femoral sections were de-waxed in xylene and rehydrated in ethanol and water. After equilibration in 0.2 M sodium acetate and 50 mM sodium tartrate, pH 5.0, for 20 min at room temperature, sections were incubated at 37°C in the same buffer containing 0.5 mg/mL naphthol AS-MX phosphate (Sigma, St Louis, MO) and 1.1 mg/mL Fast Red TR salt (Sigma, St Louis, MO) for 40 min, or until osteoclasts were bright red. Sections were then counterstained in methyl green (Sigma, St Louis, MO). Stained sections were observed under a microscope and TRAP positive cells with three or more nuclei were counted as osteoclastic cells. Femur distal epiphyseal osteoclasts were quantified by using a method modified from Sawyer et al [58].

### siRNA transfection and quantification of protein and mRNA levels

Osteocytic MLO-Y4 cells were transfected with either Cx43 or scramble control siRNA as previously described [59]. Forty-eight hours after transfection, protein and total RNA was collected. Cx43 protein level was quantified by western blot analysis as previously described [60], except Cx43 antibody (Sigma, 1∶8000 dilution) was used. Cx43, RANKL and OPG mRNA levels were assessed, by real-time RT-PCR [61], in MLO-Y4 cells treated with either Cx43 siRNA, or scramble control, and in primary osteoblasts from wild type and Cx43 deficient mice.

### In vivo mechanical loading

Initial studies indicated that Cx43 deficient mice demonstrated an osteopenic cortical bone phenotype with expanded endocortical and periosteal surfaces compared to wild type littermates. This morphology suggests an accumulation of elevated periosteal osteoblast function and elevated endocortical osteoclast function through development. We confirmed this morphologic phenotype in 17 week old mice via high resolution μCT. Concurrent bone labeling indicated that the periosteal surface was highly active in the majority of Cx43 deficient mice at this age.

As an active periosteum has the potential to confound assessment of bone's response to physiologic loading [62], we initiated the loading protocol when mice were 6 months of age. At this time, dynamic histomorphometry of sentinel mice indicated minimal baseline periosteal bone formation rate (p.BFR) in both Cx43 deficient and wild type mice. Three days prior to initiation of the loading protocol, the right tibia mid-diaphysis of all mice were imaged via high resolution μCT (Scanco vivaCT40) and custom algorithms were used to develop animal specific estimates for normal strains induced by our non-invasive tibia loading device [63]. We altered applied end loads such that peak induced compressive normal strains at the tibia mid-shaft in Cx43 deficient mice (n = 7; 1300±90 μstrain) were not statistically different than those of wild type mice (n = 7; 1275±90 μstrain p = 0.83). Each mouse underwent a 3 week, 3 day/week loading intervention, in which the mouse was anesthetized, the right tibia placed in the loading device, and the tibia loaded for 100 cycles per bout at 1 Hz. calcein labels were injected on day 10 and 19, and following sacrifice on day 21, mid-shaft cross-sections from left (contralateral) and right (experimental) tibiae were processed for dynamic histomorphometry. As we equilibrated exogenously induced strains on the periosteal surface, we confined our analysis to that surface. Mineralizing surface (p.MS), mineral apposition rate (p.MAR) and bone formation rate (p.BFR) were quantified using standard techniques.

### Statistical Analyses

Data are expressed as means ± SD. Statistical significance was assessed by Student's t-test when comparing two groups. One way ANOVA was used to assess multiple group comparisons and a Tukey post-hoc test to assess differences between groups. p<0.05 was considered significant.
